# Stimulating Factors and Origins of Precursor Cells in Traumatic Heterotopic Ossification Around the Temporomandibular Joint in Mice

**DOI:** 10.3389/fcell.2020.00445

**Published:** 2020-06-18

**Authors:** Yan Zhao, Ningjuan Ouyang, Long Chen, Hanjiang Zhao, Guofang Shen, Jiewen Dai

**Affiliations:** ^1^Department of Oral & Cranio-maxillofacial Science, Shanghai Ninth People’s Hospital, Shanghai Jiao Tong University School of Medicine, Shanghai Key Laboratory of Stomatology, National Clinical Research Center for Oral Disease, Shanghai, China; ^2^Department of Orthodontics, Shanghai Ninth People’s Hospital, Shanghai Jiao Tong University School of Medicine, Shanghai, China

**Keywords:** heterotopic ossification, temporomandibular joint, trauma, precursor cells, TGFβ2

## Abstract

The contributing factors and the origins of precursor cells in traumatic heterotopic ossification around the temporomandibular joint (THO-TMJ), which causes obvious restriction of mouth opening and maxillofacial malformation, remain unclear. In this study, our findings demonstrated that injured chondrocytes in the condylar cartilage, but not osteoblasts in the injured subchondral bone, played definite roles in the development of THO-TMJ in mice. Injured condylar chondrocytes without articular disc reserves might secrete growth factors, such as IGF1 and TGFβ2, that stimulate precursor cells, such as endothelial cells and muscle-derived cells, to differentiate into chondrocytes or osteoblasts and induce THO-TMJ. Preserved articular discs can alleviate the pressure on the injured cartilage and inhibit the development of THO-TMJ by inhibiting the secretion of these growth factors from injured chondrocytes. However, the exact molecular relationships among trauma, the injured condylar cartilage, growth factors such as TGFβ2, and pressure need to be explored in detail in the future.

## Introduction

Heterotopic ossification (HO) is a kind of pathological osteogenesis that often develops in the soft tissue around large joints, such as the hip, shoulder, and temporomandibular joint (TMJ; Ranganathan et al., 2015). Trauma is one of the most crucial factors that contribute to HO. Many kinds of injury around the joint, such as burns around the elbow or shoulder and even spinal cord injury, can induce the development of HO (Barfield et al., 2017). Some previous studies showed that trauma induced responses and changes in the local microenvironment, which may stimulate some precursor cells to participate in the induction of ectopic chondrogenesis or osteogenesis (Dey et al., 2017); in addition, local oxygen concentrations decrease after trauma, which could promote the expression of Hif-1α and Vegf-α, followed by induction of angiogenesis. Then, an abundant blood supply may bring enough chondro-osteogenic precursors to the site of injury, where HO will gradually develop around the injured joint (Agarwal et al., 2016). Studies have shown that many growth factors secreted by different cells may play important roles during the development of HO (Reichel et al., 2014), and members of the transforming growth factor superfamily, such as TGFβ2 and BMP4, have been proven to contribute to fibrodysplasia ossificans progressiva (FOP), a genetic form of HO, by promoting the transformation of precursors into chondrocytes or osteoblasts (Yu et al., 2008). Endothelial cells, mesenchymal stem cells, tendon progenitor cells, and other cells have been demonstrated to have the ability to participate in the initiation of HO. However, whether there are differences in precursor cell origins among different joints or different conditions is not clear (Xu et al., 2018).

Traumatic heterotopic ossification around the temporomandibular joint (THO-TMJ) may lead to ankylosis of the TMJ in some patients (Meng et al., 2013), which not only limits the degree to which the mouth can open but also causes serious cranio-maxillofacial development deformities (He et al., 2014). The pathogenic mechanism of THO-TMJ is complicated, and immune-inflammatory responses caused by trauma in the local microenvironment may disrupt the balance between osteoblasts and osteoclasts around the TMJ (Shehab et al., 2002; Dai et al., 2016). A previous study proposed that a hematoma in the joint capsule may be the initial factor that causes THO-TMJ. In addition, some researchers proposed that the effect of distraction osteogenesis of the lateral pterygoid muscle may be a contributing factor to this process (Meng et al., 2009). In our previous study and our combined clinical experience, we preliminarily found that injured condylar cartilage but not injured bone in the TMJ may play a crucial role in causing THO-TMJ (Zhao et al., 2019). Some studies have shown that chondrocytes have autocrine and paracrine functions, especially secondary to inflammation, trauma, or changes in pressure and pH (Hinton et al., 2017). However, whether and how the residual chondrocytes in the injured condylar cartilage of the TMJ, in comparison to osteoblasts in the injured bone or normal chondrocytes, secrete additional growth factors, such as TGFβs or BMPs, that might contribute to THO-TMJ is not clear. In addition, the TMJ discs are crucial to physiological function and even some pathological processes, so whether the articular disc influences the pathological process of THO-TMJ also needs to be investigated.

In this study, we constructed animal models to identify and verify the influencing factors of the pathological process of THO-TMJ, including the origins of precursor cells in THO-TMJ, the growth factors secreted by the injured condylar cartilage but not the subchondral bone, and the role of the articular discs during the pathological process of THO-TMJ.

## Materials and Methods

### Animal Models

Four-week-old male mice with C57BL/6J genetic background were used in this study. Animal experimental procedures were performed in compliance with the Institutional Animal Care and Use Committees of the Shanghai Ninth People’s Hospital, Shanghai Jiao Tong University School of Medicine. The procedures were reviewed and approved by the Ethics Committees of the Shanghai Ninth People’s Hospital, Shanghai Jiao Tong University School of Medicine, China (approval number 2016-45).

**FIGURE 1:**
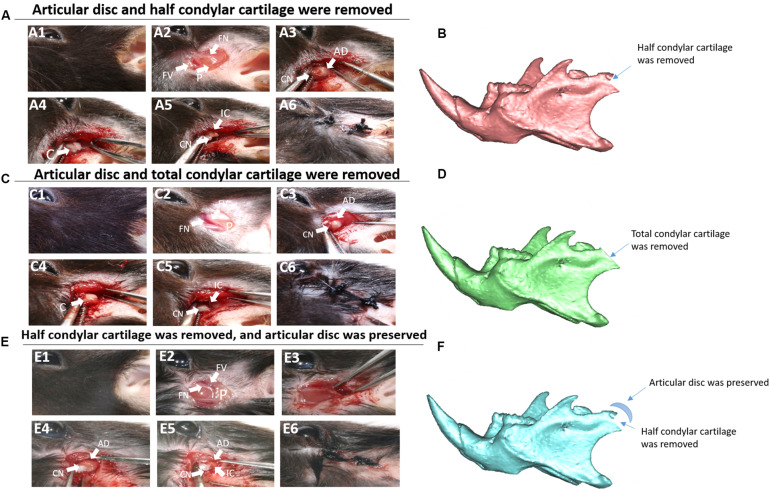
Animal model construction. **(A)** The first animal model (the articular disc and half condylar cartilage were removed in mice). **(A1)** Surgery on the left side. **(A2)** The preauricular skin was cut, and the facial vessels and nerves were exposed. **(A3)** The muscle was cut, and the integral condyle was exposed. **(A4)** The articular capsule and disc were removed. **(A5)** The latter half of the condylar cartilage was removed using scissors. **(A6)** The wound was closed. **(B)** 3D schematic of the first animal model. **(C)** The second animal model (the articular disc and total condylar cartilage were removed in mice). **(C1–C4,C6)** show the same procedures described above. **(C5)** Total condylar cartilage was removed using scissors. **(D)** 3D schematic of the second animal model. **(E)** The third animal model (half condylar cartilage was removed, but the articular disc was preserved). **(E1–E3,E6)** show the same procedures described above. **(E4)** The articular disc was preserved. **(E5)** The latter half of the condylar cartilage was removed using scissors, ensuring that there was no evident displacement of the articular disc. **(F)** 3D schematic of the third animal model. AD, articular disc; C, condyle; CN, condyle neck; FN, facial nerve; FV, facial vessel; IC, injured condyle; P, parotid gland.

In this study, we established three related animal models (Figure 1): (1) The first animal model: articular disc and half condylar cartilage were both removed in the left mandibular condyle of mice with small scissors to induce THO-TMJ. (2) The second animal model: articular disc and all condylar cartilage were completely removed in the left joint in mice. (3) The third animal model: half condylar cartilage was removed but the articular disc was preserved. The protocol for establishment of these mouse models was described as follows: briefly, 4-week male mice were anesthetized with 0.8% pentobarbital sodium intraperitoneally. Surgery was performed on the left mandibular joint, whereas the right side was used as the control. The preauricular skin was sterilized with 75% alcohol, and a 0.8-cm linear preauricular skin incision was made. The preauricular fascia was cut open just above the superior border of the facial vessel to expose the zygomatic arch. Then, the joint capsule was cut open to expose the condyle, and half of the condylar cartilage was then removed using small scissors in the first model, whereas the condylar cartilage was completely removed in the second animal model. Both in the first and second animal model, the joint capsule was opened, and the articular disc was removed. In the third model, the latter part of the condylar cartilage was exposed and removed, but the joint capsule needed to be peeled lightly to preserve the articular disc. Finally, the incision was closed using a 3-0 suture. The mice were cared for under a 37°C environment after surgery and given soft food for 2 weeks.

To trace the origins of precursor cells in THO-TMJ, Tie2-Cre mice on the C57BL/6J genetic background were bred with Rosa26-Lacz^flox/flox^ mice to obtain Tie2-Cre/Lacz^flox/flox^ mice for tracing endothelial cells, and Ckmm-Cre mice were bred with Rosa26-Lacz^flox/flox^ mice to obtain Ckmm-Cre/Lacz^flox/flox^ mice for tracing muscle-derived cells during the development of THO-TMJ. All mice were obtained from the Shanghai Research Center of Southern Model Organisms (Shanghai, China). We removed the articular discs and half of the condylar cartilage in Tie2-Cre/Lacz^flox/flox^ mice or Ckmm-Cre/Lacz^flox/flox^ mice to generate the THO-TMJ model, which helped us trace the origins of precursor cells in THO-TMJ.

### Radiological Examination and Imaging Analysis

#### Micro-CT Scans

Mice were anesthetized intraperitoneally with pentobarbital sodium before micro-CT scans were performed. The slice thickness for the micro-CT scans was 40 μm using an eXplore Locus micro-CT scanner (GE Healthcare, Milwaukee, WI, United States). Three-dimensional (3D) reconstruction of the skulls was performed using GE MicroView software (GE Healthcare, Milwaukee, WI, United States). The bone volume was measured using GeomagicStudio software (Geomagic, NC, United States).

#### Micro-MRI Examination

Mice were anesthetized using 1.5% isoflurane mixed with air. Then, the mice were placed in the sample tube in the prone position. The magnetic field intensity was 7.0 T, and the main frequency was 300 MHz using a Bruker Bioapec 70/20 USR scanner (Bruker, Germany). The image information was acquired and analyzed using the Athena DICOM Viewer.

#### Micro-SPECT Examination

The mice were anesthetized using 1.5% isoflurane mixed with air and then placed in the sample tube in the prone position. 99mTc-MDP was used as an imaging probe, with 3 mCi injected into every mouse via the tail vein. The energy peak was set as 140 keV, and the bandwidth was set as 20% using a Bioscan Nano SPECT/CT plus scanner (Bioscan, United States). The image information was acquired and analyzed with ImageJ software, and then the tissue metabolism was compared between the injured side and control side.

### Histological Analysis

Whole heads of mice at indicated time points were freely dissected and collected, and the samples were fixed in 4% paraformaldehyde and demineralized in 0.5 M ethylenediaminetetraacetic acid for 2–3 weeks in the 4°C environment. Then, the samples were embedded in paraffin and cut to 5 μm thickness for regular hematoxylin and eosin (H&E) staining and to 4 μm thickness for safranin O (Sigma-Aldrich, S2255) and fast green (Sigma-Aldrich, F7252) staining. Briefly, for safranin O staining, slides were incubated in safranin O solution for 5–10 min, which was determined by the degree of orange. Then, the slides were washed in differentiation solution and stained with fast green solution for at least 10 min at 37°C. Subsequently, the stained slides were washed with running tap water and cleared in xylene. Finally, the slides were mounted with resinous mounting medium for observation and image acquisition through inverted microscope.

### Immunofluorescence Costaining

Whole heads of mice of different animal models were dissected and collected seriously and then were embedded in paraffin after washing, fixation, and demineralization as previously stated. Subsequently, the tissue samples were cut to 5 μm thickness and put them on the slide warmer, which was at least 60°C for 2 h. The slides were then deparaffinized with xylene twice and hydrated with gradient alcohol. Next, antigen retrieval was performed using an antigen restoration liquid kit (Sunteam Biotech, China). For the immunofluorescence staining, the slides were subsequently blocked with 5% donkey serum in phosphate-buffered saline (PBS) for 1 h and incubated with anti-IGF1 (Abcam, 1/300), anti-TGFβ2 (Abcam, 1/150), or anti-TRPV4 (Abcam, 1/300) combined with anti-Osterix (Abcam, 1/300) or anti-Sox9 (Abcam, 1/300) primary antibodies overnight at 4°C. Subsequently, secondary antibodies (AlexaFluor 568 donkey anti-mouse and AlexaFluor 488 donkey anti-rabbit, Jackson) were diluted in PBS (1/400) and incubated at room temperature for 1 h.

In addition, for immunofluorescence staining for cell lineage tracing, the slides were incubated with anti-Sox9 (Abcam, 1/300) and anti-β-galactosidase (Abcam, 1/200) primary antibodies overnight at 4°C after blocking with 5% donkey serum in PBS for 1 h. Furthermore, secondary antibodies (AlexaFluor 568 donkey anti-chicken and AlexaFluor 488 donkey anti-rabbit, Jackson) were diluted in PBS (1/400) and incubated at room temperature for 1 h. Finally, all of the slides were mounted with fluorescence-protecting glycerol containing DAPI (Solarbio, China) for visualization under a fluorescence microscope.

### Quantitative PCR

Tissues were harvested from the residual condylar cartilage/subchondral bone of mice, or from normal cartilage/subchondral bone of mice as the control group at indicated time points. Total RNA was collected from tissues using trizol reagent and fully grinding tissues with dry ice. Reverse transcription was performed with 1 μg RNA using Reverse Transcription Reagents (Takara, Japan). Quantitative real-time PCR was carried out using SYBR Green Premix Quantitative PCR reagents (Takara, Japan) and StepOnePlus Real-Time PCR System (Applied Biosystems, United States). Specific primers for these genes were chosen based on their PrimerBank sequence:

(1) Gapdh, **F:**CAGGAGAGTGTTTCCTCGTCC,**R:**TTTGCC GTGAGTGGAGTCAT;
(2) Igf1, **F:**GCTCTGCTTGCTCACCTTCACC,**R:**ACACTC ATCCACATGCCTGTCTG;
(3) Tgfβ2, **F:**GGCAGATCCTGAGCAAGCTGAAG,**R:**AACC TCCTTGGCGTAGTACTCCTC.

### Statistical Analysis

The data are expressed as the mean ± SEM. Data analysis (*n* ≥ 3) was performed using one-way ANOVA with SPSS 18.0 software (International Business Machines, Armonk, NY, United States). *p* < 0.05 was considered to indicate a statistically significant difference.

## Results

### Results of Radiological Examination of the Role of Injured Condylar Cartilage in the Development of THO-TMJ

#### Micro-CT Examination

**FIGURE 2:**
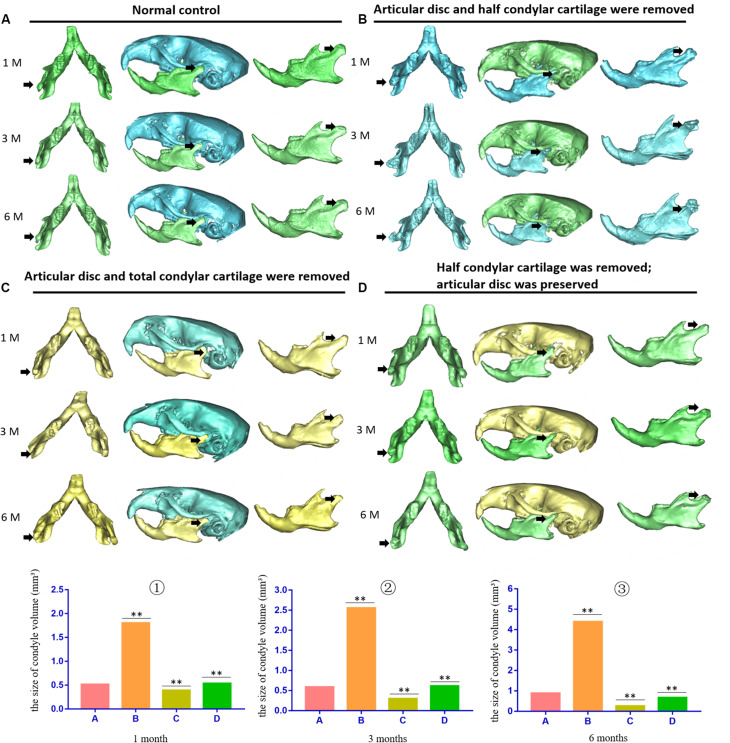
3D micro-CT of the skull and mandible of different animal models during the development of TMJ-THO. **(A)** Normal control TMJ of mice at the same time points after surgery. **(B)** TMJ structure of the first animal model (the articular disc and half condylar cartilage were removed) at different time points. Heterotopic ossification was obvious around the injured TMJ at different time points. In addition, the adhesion between the condyle and surrounding tissues also progressed over time. **(C)** TMJ structure of the second animal model (the articular disc and total condylar cartilage were removed) at different time points. There was no evident heterotopic ossification or adhesion around the TMJ. **(D)** TMJ structure of the third animal model (half of the condylar cartilage was removed, but the articular disc was preserved) at different time points. There was also no obvious heterotopic ossification or adhesion around the TMJ. The black arrow indicates the injured side of the TMJ. ①, ②, and ③ showed that the volume of condyle in the first animal model was higher than that of normal condyle due to the formation of heterotopic ossification; however, the volume of condyle in the second or in the third animal model was lower than that of the first animal model at different time points. A, the control groups; B, the first animal model; C, the second animal model; D, the third animal model, ^∗∗^*p* < 0.01. 1 M, 1 month after surgery; 3 M, 3 months after surgery; 6 M, 6 months after surgery.

The pathological changes at 1, 3, and 6 months after establishment of the animal model were evaluated. In the first animal model (the articular disc and half condylar cartilage were removed), the 3D skull reconstructed from micro-CT images showed that removing half of the condylar cartilage and the articular disc led to obvious development of HO around the injured TMJ compared with the contralateral healthy TMJ. The shape of the injured TMJ and the adhesion around the injured condylar region also changed over time and exhibited aggravation. The ectopic tissues and injured condyle fused to become an osteophyte, which led to a gradual increase in the volume of the condyle. However, in the second model (the articular disc and total condylar cartilage were removed), removing all of the condylar cartilage and the articular disc did not result in obvious HO around the injured TMJ, but the condyle lost its original shape compared with that of the normal condyle on the contralateral side. Moreover, in the third model (half of the condylar cartilage was removed with the articular disc preserved), there was also no obvious ectopic bone around the injured condyle over time (Figure 2). According to the comparison between the volume of normal condyle and the volume of injured condyle, we found that about 80% (12 out of 15 mice) of the animals in the first model (the articular disc and half condylar cartilage were removed) could develop HO 3 months after the surgery; however, only 1 out of 15 mouse in the second model (the articular disc and all condylar cartilage were removed) exhibited HO, and 2 out of 15 mice in the third model (half of the condylar cartilage was removed with the articular disc preserved) exhibited HO.

#### Micro-MRI and Micro-SPECT Examination

**FIGURE 3:**
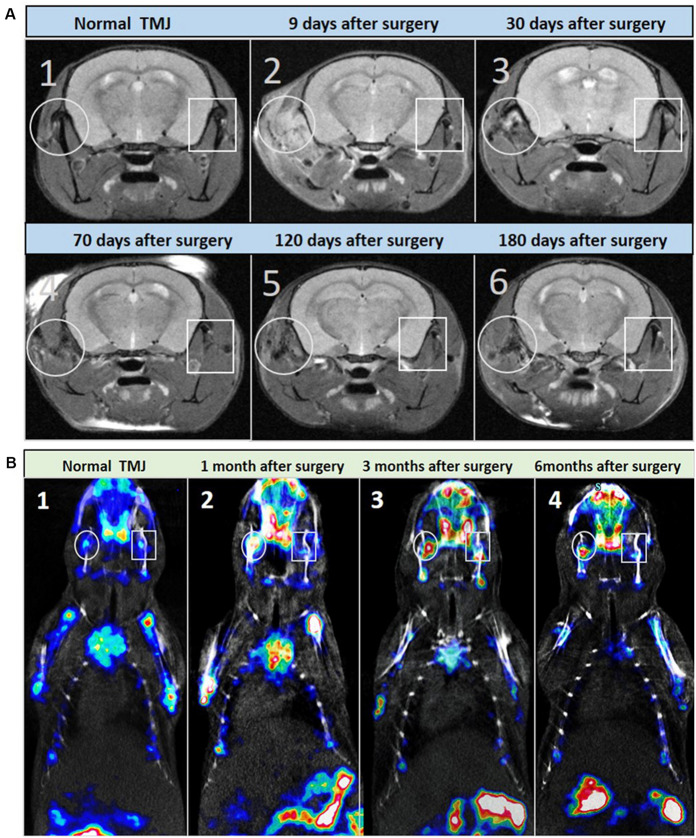
Micro-MRI and micro-SPECT examination of the first animal model (the articular disc and half condylar cartilage were removed) during the development of THO-TMJ. **(A)** Micro-MRI examination results showed a strong T2 signal around the TMJ on the injured side, which was gradually reduced and replaced by a weak signal of heterotopic ossification compared with the normal side of the TMJ in the animal model **(A2–A6)** and the TMJ in the normal control mice **(A1)** over time. **(B)** Micro-SPECT examination results showed no obvious difference in bone metabolism between the two sides of the TMJ in normal control mice **(B1)**, but it showed strong bone metabolism (red color) in the injured TMJ region compared with that in the normal side at different time points after surgery **(B2–B4)**; white circle: left, injured TMJ; white rectangle: right, normal TMJ.

The pathological changes of THO-TMJ were further confirmed in the first animal model (the articular disc and half condylar cartilage were removed) through micro-MRI examination at different time points. The T2-weighted image demonstrated a strong signal around the TMJ region on the injured side, which indicated inflammatory edema in the initial stage of trauma. However, the strong signal gradually became weaker over time due to the gradual development of HO (weak signal) around the TMJ. In the latter stage, the normal structure of the TMJ was replaced by an osteophyte formed by fusion of the injured condyle and the ectopic tissue around it, and the T2-weighted image revealed a weaker signal around the TMJ region on the injured side than on the normal side (Figure 3A).

The metabolism of HO in the first animal model was also evaluated through micro-SPECT examination, and the results showed that there was stronger bone metabolism in the traumatic TMJ region than in the contralateral healthy TMJ at different time points (Figure 3B).

### Results of qPCR for the mRNA Expression of IGF1 and TGFβ2 in the Injured Condylar Cartilage of the First Animal Model and in the Injured Subchondral Bone of the Second Animal Model

**FIGURE 4:**
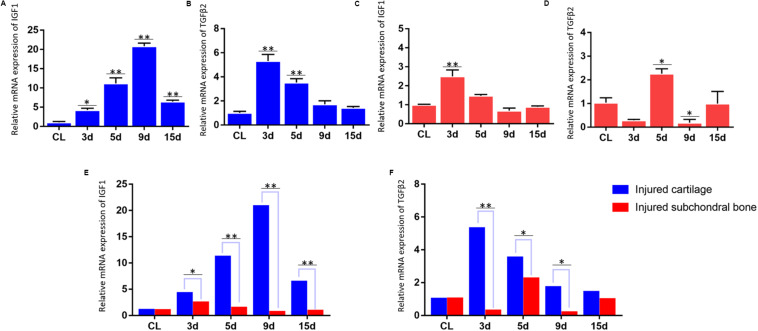
qPCR results for the mRNA expression of IGF1 and TGFβ2. **(A,B)** Dynamic changes in IGF1 and TGFβ2 mRNA expression in the injured TMJ region at different time points in the first animal model (the articular disc and half condylar cartilage were removed) compared with the control groups (normal cartilage). **(C,D)** Dynamic changes in IGF1 and TGFβ2 mRNA expression in the injured TMJ region at different time points in the second animal model (the articular disc and total condylar cartilage were removed) compared with the control groups (normal subchondral bone). **(E)** Comparison of the relative change of IGF1 mRNA in the injured TMJ region between the first animal model and the second animal model at different time points. **(F)** Comparison of the relative change of TGFβ2 mRNA in the injured TMJ region between the first animal model and the second animal model at different time points. Abbreviation: CL, control; 3 d, 5 d, 9 d, and 15 d indicate 3 days, 5 days, 9 days, and 15 days, respectively; ^∗^*p* < 0.05, ^∗∗^*p* < 0.01.

Differential expression of growth factors in the injured cartilage in the first animal model (the articular disc and half condylar cartilage were removed) and the injured subchondral bone in the second animal model (the articular disc and total condylar cartilage were removed) was evaluated in this study, and the qPCR results showed that the expression of Igf-1 and Tgf-β2 increased significantly compared with that of the normal cartilage in the first animal model at different time points (Figures 4A,B). However, the residual subchondral bone in the second animal model showed no obvious increase in the expression of these two growth factors at most time points and even decreased at some time points (Figures 4C,D). In addition, the relative change of expression of Igf-1 and Tgf-β2 in the injured cartilage in the first animal model was significantly higher than that in the subchondral bone in the second animal model (Figures 4E,F).

### Immunofluorescence Costaining for the Expression of IGF1 and TGFβ2 in the TMJ Region in Different Animal Models

**FIGURE 5:**
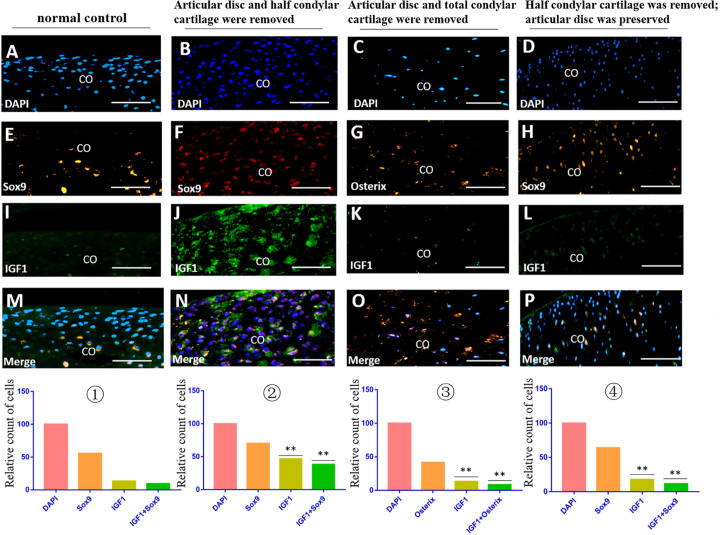
IGF1/Sox9 and IGF1/Osterix immunofluorescence double staining in the TMJ in different animal models. **(A–D)** DAPI staining. **(E,F,H)** Sox9 staining. **(G)** Osterix staining. **(I–L)** IGF1 staining. **(M–P)** Merged image of DAPI, Sox9/Osterix, and IGF1 staining. ① The cell counts of different markers in the normal TMJ, with the count of DAPI accepted as 100 and the counts of other markers calculated and compared with the count of DAPI. ② The relative counts of IGF1-positive cells and costained cells were higher in the injured condylar cartilage in the first animal model than those in the normal TMJ (^∗∗^*p* < 0.01). ③ The relative counts of IGF1-positive cells and costained cells were lower in the injured subchondral bone in the second animal model than those in the first animal model (^∗∗^*p* < 0.01). ④ The relative counts of IGF1-positive cells and costained cells were also lower in the injured condylar cartilage in the third animal model than those in the first animal model (^∗∗^*p* < 0.01). AD, articular disc; CO, condyle. Scale bar: 50 μm.

**FIGURE 6:**
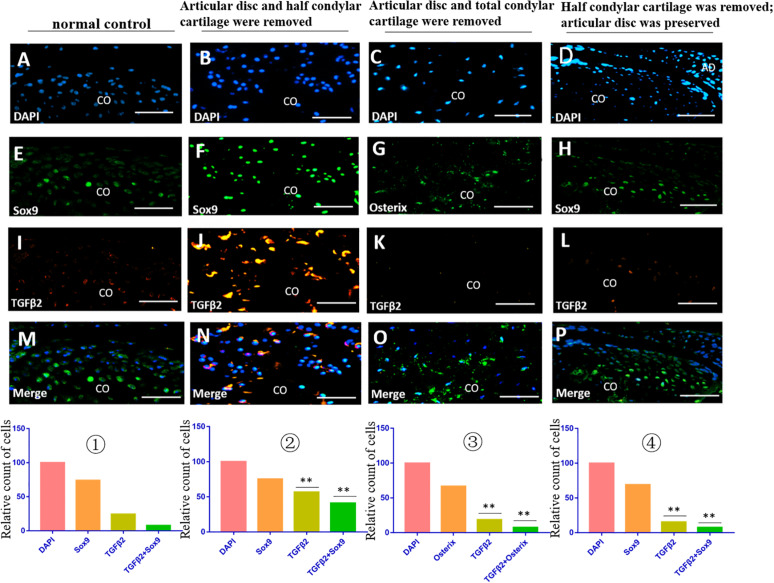
TGFβ2/Sox9 and TGFβ2/Osterix immunofluorescence double staining in the TMJ in different animal models. **(A–D)** DAPI staining. **(E,F,H)** Sox9 staining. **(G)** Osterix staining. **(I–L)** TGFβ2 staining. **(M–P)** Merged image of DAPI, Sox9/Osterix, and TGFβ2 staining. ① The cell counts of different markers in the normal TMJ, with the count of DAPI accepted as 100 and the counts of other markers calculated and compared with the count of DAPI. ② The relative counts of TGFβ2-positive cells and costained cells were higher in the injured condylar cartilage in the first animal model than those in the normal TMJ (^∗∗^*p* < 0.01). ③ The relative counts of TGFβ2-positive cells and costained cells were lower in the injured subchondral bone in the second animal model than those in the first animal model (^∗∗^*p* < 0.01). ④ The relative counts of TGFβ2-positive cells and costained cells were also lower in the injured condylar cartilage in the third animal model than those in the first animal model (^∗∗^*p* < 0.01). AD, articular disc; CO, condyle. Scale bar: 50 μm.

The immunofluorescence costaining results showed that there were obvious TGFβ2/Sox9 and IGF1/Sox9 double-positive chondrocytes in the residual injured condylar cartilage in the first animal model (the articular disc and half condylar cartilage were removed) compared with the normal control group. However, there were no obvious TGFβ2/Osterix and IGF1/Osterix double-positive osteoblasts in the residual injured subchondral bone in the second animal model (the articular disc and all condylar cartilage were removed), and there were also fewer TGFβ2/Sox9 and IGF1/Sox9 double-positive chondrocytes in the injured condylar cartilage of the third animal model (half condylar cartilage was removed with the articular disc preserved) (Figures 5, 6).

### H&E, Safranin O, and Fast Green Staining Results for the Role of the Condylar Cartilage and the Articular Disc in the Development of THO-TMJ

**FIGURE 7:**
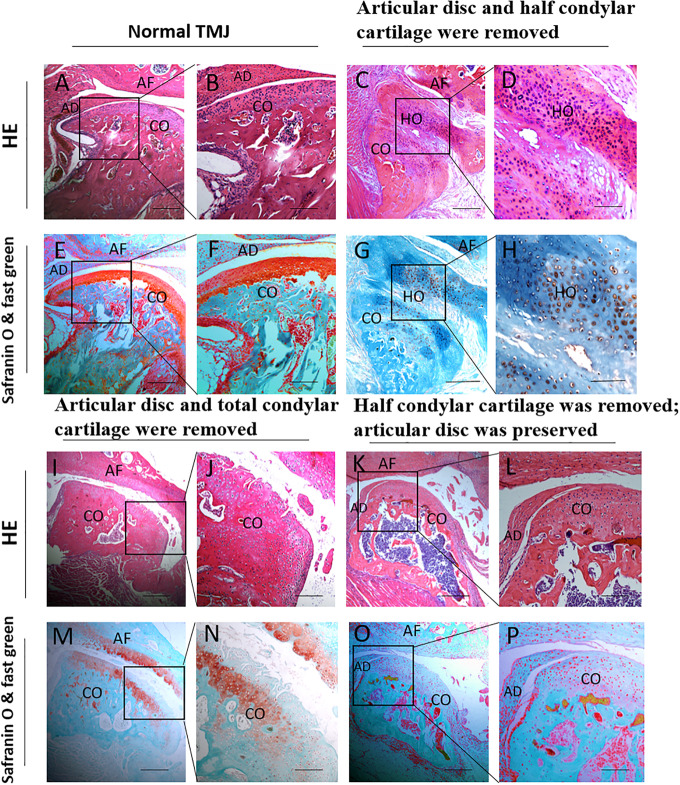
Histological examination of different animal models during the development of TMJ-THO 3 months after surgery. **(A,B,E,F)** The TMJ structure in normal control mice. **(C,D,G,H)** There was obvious heterotopic ossification around the injured TMJ, and the structure of the condyle showed an abnormal shape in the first animal model (the articular disc and half condylar cartilage were removed). **(I,J,M,N)** No obvious heterotopic ossification around the injured condyle in the second animal model (the articular disc and total condylar cartilage were removed). **(K,L,O,P)** No obvious heterotopic ossification around the injured condyle was observed in the third animal model (half of the condylar cartilage was removed, but the articular disc was preserved). Panels **(B,D,F,H,J,L,N,P)** are local magnifications of the boxed areas in panels **(A,C,E,G,I,K,M,O)**, respectively. AD, articular disc; AF: articular fossa; CO, condyle; HO, heterotopic ossification. Scale bar: **(A,C,E,G,I,K,M,O)**, 200 μm; **(B,D,F,H,J,L,N,P)**, 50 μm.

In the first animal model (the articular disc and half condylar cartilage were removed), the H&E, safranin O, and fast green staining results showed obvious HO around the injured condyle, with an abnormal histological structure after H&E staining. In addition, the safranin O and fast green staining results showed that the HO involved endochondral ossification, in which the glaucous bone surrounded the orange cartilage. However, there was no ectopic cartilage or bone formation around the TMJ in the second animal model (the articular disc and total condylar cartilage were removed) or the third animal model (half of the condylar cartilage was removed with the articular disc preserved), and the general condylar structure in these two groups could be recognized (Figure 7).

### Immunofluorescence Costaining for the Expression of TRPV4 in the TMJ Region in Different Animal Models

**FIGURE 8:**
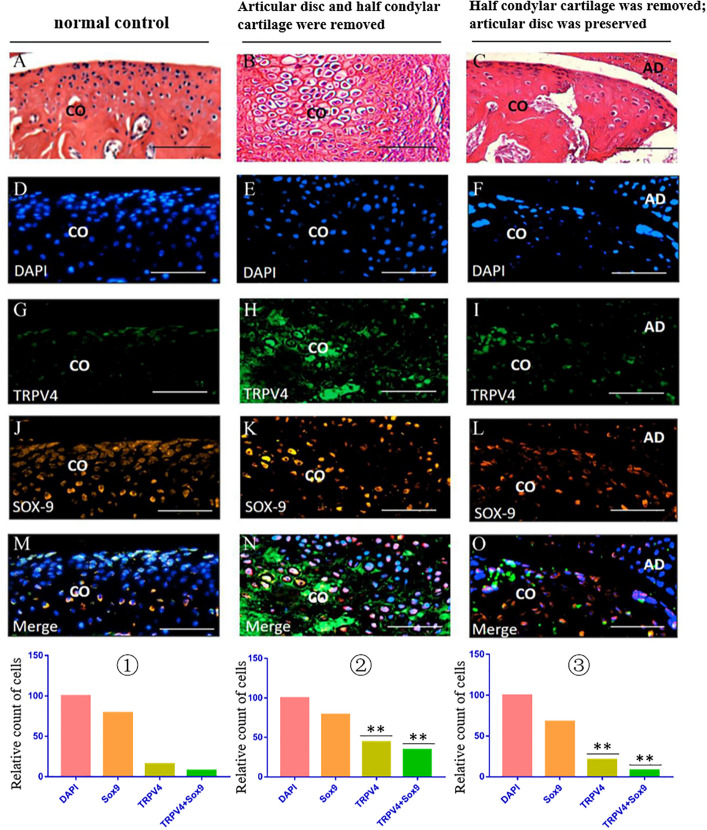
TRPV4/Sox9 immunofluorescence double staining in the TMJ in different animal models. **(A–C)** H&E examination revealed the structure of TMJ in different animal models. **(D–F)** DAPI staining. **(G–I)** TRPV4 staining. **(J–L)** Sox9 staining. **(M–O)** Merge of DAPI, Sox9, and TRPV4 staining. ① The cell counts of different markers in the normal TMJ, with the count of DAPI accepted as 100 and the counts of other markers calculated and compared with the count of DAPI. ② The relative counts of TRPV4-positive cells and TRPV4/Sox9 costained cells were higher in the injured condylar cartilage in the first animal model than in the normal TMJ (^∗∗^*p* < 0.01). ③ The relative counts of TRPV4-positive cells and TRPV4/Sox9 costained cells were lower in the injured condyle cartilage in the third animal model than in the first animal model (^∗∗^*p* < 0.01). AD, articular disc; CO, condyle. Scale bar: 50 μm.

The immunofluorescence costaining results showed that there were obvious TRPV4/Sox9 double-positive chondrocytes in the injured condylar cartilage in the first animal model (articular disc and half condylar cartilage were removed) when compared with the normal control group. However, there were fewer TRPV4/Sox9 double-positive chondrocytes in the residual injured condylar cartilage in the third animal model (half condylar cartilage was removed with articular disc preserved) (Figure 8).

### Precursor Cell Linage Tracing in THO-TMJ

**FIGURE 9:**
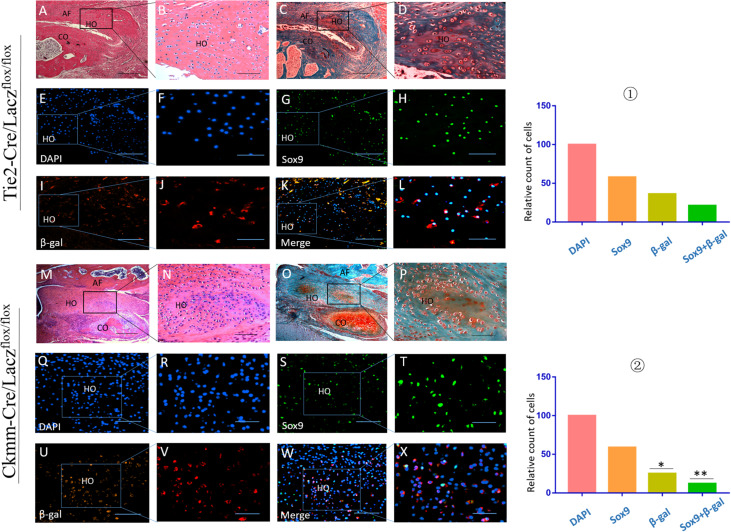
Precursor cell lineage tracing in THO-TMJ. H&E examination **(A,B,M,N)** and safranin O and fast green examination **(C,D,O,P)** revealed the structure of the TMJ, which suffered from obvious HO. **(E–L)** Precursor cell lineage tracing in heterotopic ossification tissues in the Tie2-Cre/Lacz^flox/flox^ THO-TMJ mouse model. **(Q–X)** Precursor cell lineage tracing in heterotopic ossification tissues in the Ckmm-Cre/Lacz^flox/flox^ THO-TMJ mouse model. **(E,F,Q,R)** DAPI staining. **(G,H,S,T)** Sox9 staining. **(I,J,U,V)** Immunofluorescence staining of β-gal. **(K,L,W,X)** Merged images of DAPI, Sox9, and β-gal. ① The relative cell counts of different markers in Tie2-Cre/Lacz^flox/flox^ THO-TMJ mice, with the count of DAPI regarded as 100 and the counts of other markers calculated and compared with the count of DAPI. ② The relative cell counts of different markers in Ckmm-Cre/Lacz^flox/flox^ THO-TMJ mice showed fewer β-gal-positive cells and β-gal/Sox9 costained cells than observed in Tie2-Cre/Lacz^flox/flox^ THO-TMJ mice (*p < 0.05, **p < 0.01). Panels **(B,D,F,H,J,L,N,P,R,T,V,X)** are local magnifications of the boxed areas in panels **(A,C,E,G,I,K,M,O,Q,S,U,W)**. AF, articular fossa; CO, condyle; HO, heterotopic ossification. Scale bar: **(A,C,M,O)**, 200 μm; **(B,D,E,G,I,K,N,P,Q,S,U,W)**, 50 μm; **(F,H,J,L)**, 25 μm; **(R,T,V,X)**, 20 μm.

Tie2-Cre/Lacz^flox/flox^ and Ckmm-Cre/Lacz^flox/flox^ mice were used to generate the THO-TMJ animal model by removing the articular disc and half of the condylar cartilage. Immunofluorescence costaining showed that there were cells that expressed both Sox9 and β-galactosidase (β-gal), as indicated by Lacz in the HO region in these two mouse strains, which showed that some precursor cells of THO-TMJ originated from endothelial cells and muscle-derived cells. Furthermore, the number of costained cells in the Tie2-Cre/Lacz^flox/flox^ group was larger than that in the Ckmm-Cre/Lacz^flox/flox^ group, but the total number of double-positive cells in these two mouse strains was smaller than that in the Sox9-positive cells, which showed that there might be other precursor cells that contribute to THO-TMJ (Figure 9). We also performed positive and negative control groups for the lineage tracing animal models (Supplementary Figure 1).

## Discussion

The TMJ is one of the most complex joints and is composed of the temporal articular surface, condyle, articular disc, articular capsule, synovium and articular ligament, as well as closely associated with the lateral pterygoid muscle (Schmolke, 1994). Every part of the TMJ performs its own function, and the components become a functional complex to perform the processes of mastication, pronunciation, and deglutition (Cimic et al., 2015). However, due to its exposed location and high frequency of motion, the TMJ easily suffers from all kinds of trauma, and sagittal fracture of the condyle is a strong risk factor for HO around the TMJ, which may be correlated with residual chondrocytes in the injured cartilage (Yan et al., 2014). In this study, the results further showed that there was obvious HO around the injured TMJ when the articular disc and half of the condylar cartilage were both removed, and the development process was proven through various imaging methods. However, there was no obvious HO around the injured TMJ when all of the condylar cartilage and the articular disc were removed, which further indicated that injured cartilage but not subchondral bone is an important contributing factor related to the THO-TMJ pathological process.

The articular disc is an essential structure of the TMJ and is associated with many TMJ diseases due to its important physiological functions, such as coordinating structural differences between the temporal articular surface and the condyle, dispersing strength, and alleviating masticatory pressure (Li et al., 2006; Pihut et al., 2017). In addition, the articular disc also serves as a barrier to divide the articular cavity into the upper part and lower part (Detamore and Athanasiou, 2003). In this study, the results showed that there was no obvious HO around the injured TMJ when half of the condylar cartilage was removed but the articular disc was preserved. In addition, the shape of the condyle also did not show obvious degenerative changes with the articular disc was preserved. These findings implied that the articular disc has an inhibitory effect on THO-TMJ. We also found that the expression of Trpv4, a pressure-mechanosensitive ion channel, also showed higher expression in the residual condylar chondrocytes without articular disc preservation than in those with articular disc preservation. It implied that the articular disc might inhibit HO formation by alleviating pressure through the biomechanical signaling pathway.

Many kinds of tissue cells in the TMJ, such as chondrocytes, osteoblasts, and synoviocytes, have complicated feedback mechanisms when the surrounding microenvironment changes (Ibi, 2019). Trauma is a serious condition that can decrease the partial pressure of oxygen, increase the regional blood volume, and even induce inflammation (Bae and Aronovich, 2018; Gugala et al., 2018). Chondrocytes are sensitive to the microenvironment and perform autocrine or paracrine functions in response to changes in regional oxygen tension, pH, micronutrients, and mechanical effects (Shen et al., 2017; Cowman et al., 2019). Growth factors from the IGF and TGFβ families play crucial roles in cartilage development and homeostasis. We speculated that they may also be important for TMJ-THO development, which begins with chondrogenesis. In this study, several factors from the IGF and TGFβ families were evaluated, and the qPCR and immunofluorescence costaining results showed that the injured chondrocytes expressed higher levels of IGF1 and TGFβ2 in the first animal model (the articular disc and half condylar cartilage were removed) than in the normal cartilage; however, these two growth factors exhibited lower expression in injured osteoblasts in the second animal model (the articular disc and total condylar cartilage were removed) than in the injured chondrocytes in the first animal model. The immunofluorescence costaining results also showed that IGF1 and TGFβ2 did not show different expression between injured cartilage in the third animal model (half condylar cartilage was removed with the articular disc preserved) and normal condyle. IGF1 is one of most important growth factors that participate in the anabolism process (Zhang et al., 2017). It can obviously promote the synthesis of cartilage matrix when homeostasis is disrupted (Boushell et al., 2019). In addition, IGF1 plays an important role in promoting cell differentiation and even contributes to phenotypic transformation of some cells with other growth factors (Reible et al., 2018). TGFβs, which belong to the transforming growth factor superfamily, also have a crucial function during the response of chondrocytes to trauma (van der Kraan, 2017). In particular, TGFβ2 has been preliminarily proven to participate in the process of HO, and the ability of TGFβ2 to transform some precursor cells into chondroblasts may play a key role (Medici et al., 2010). These findings implied that residual and injured condylar chondrocytes actively respond to trauma and secrete some growth factors that differ from those of injured subchondral osteoblasts, such as IGF1 and TGFβ2, which might promote the pathological development of THO-TMJ under traumatic conditions without the articular disc; they also showed the crucial effect of injured cartilage during HO development.

HO is a kind of pathological ossification that is usually initiated by osteogenesis or chondrogenesis precursors (Lounev et al., 2009; Downey et al., 2015). Due to microenvironmental changes caused by factors such as trauma, inflammation, and hypoxia, these precursors initiate the pathological ossification progress and transform into chondrocytes or osteoblasts (Lees-Shepard and Goldhamer, 2018). Many kinds of HO begin with chondrogenesis, followed by endochondral ossification. For example, FOP is a genetic form of HO. The cell origins of heterotopic cartilage and bone in FOP have been preliminarily proven, and some precursors differentiate into osteoblasts or chondrocytes through epithelial–mesenchymal transition (EMT) (Ramirez et al., 2014). Previous studies have shown that Scx-positive tendon-derived progenitors may mediate endochondral HO of ligaments without exogenous injury; however, the muscle-resident interstitial Mx1-positive population may be related to intramuscular injury-dependent endochondral HO (Dey et al., 2016). These findings implied that different phenotypes of HO may involve distinct precursors that transform into chondroblasts or osteoblasts. The TMJ is surrounded by synovial tissue and associated with many muscles, such as the lateral pterygoid muscle and masseter. Vascular tissue from the synovium will respond to TMJ injury and could grow into the joint space (Zhao et al., 2019). It has been found that endothelial cells from vascular tissue could transform into multipotent stem-like cells, especially under the effect of some growth factors, such as TGFβ2, and that endothelial cells could transform into chondroblasts (Medici et al., 2010). We constructed Tie2-Cre/Lacz^flox/flox^ mice and performed immunohistochemistry with antibodies specific for Sox9 to detect chondrocytes and β-gal expressed by Lacz to detect cells of endothelial origin expressing TIE2. Interestingly, cells in heterotopic chondrogenic tissue showed coexpression of β-gal with Sox9, which showed that TIE2-positive chondrocytes were derived from endothelial cells. This finding also agrees with previous studies showing that vascular tissue could embed into the injured joint space and that vascular endothelial cells could transform into chondroblasts or osteoblasts through EMT under the effect of related growth factors (Medici and Olsen, 2012). TMJ injury is often accompanied by muscle injury around the injured condyle, and cells from muscle tissue respond to homeostasis changes induced by trauma (Porter et al., 2016). Skeletal muscle is a pool of stem cells that have the characteristic of multipotential differentiation. For example, satellite cells are a kind of muscle stem cell that is crucial for the regeneration of muscle tissue (Relaix and Zammit, 2012). However, these stem cells could become multipotent in a specific microenvironment, and some growth factors could promote this process. IGF-1 plays an important role in the proliferation and differentiation of myoblasts (Witt et al., 2017). CKMM is specifically expressed in skeletal muscle tissue and can be found in muscle cells, satellite cells, and other related myoblasts (Zanou and Gailly, 2013; Mesquita-Ferrari et al., 2015). We constructed CKMM-Cre/Lacz^flox/flox^ mice to explore whether muscle-derived cells could participate in the formation of HO around the injured TMJ. In addition, we also performed immunohistochemistry with antibodies specific for Sox9 to detect chondrocytes and β-gal expressed by Lacz to detect cells of muscular origin that express CKMM. Interestingly, cells in heterotopic chondrogenic tissue showed coexpression of β-gal and SOX9, which showed that CKMM-positive chondrocytes in HO have a muscular origin. We constructed these two lineage-tracing mouse models to detect the origins of precursor cells in THO-TMJ and found not only that vascular and muscular tissues are closely related to the injured TMJ but also that endothelium-derived cells and muscle-derived cells in these two important tissues could transform into chondroblasts, followed by chondrogenesis and endochondral ossification.

## Conclusion

**FIGURE 10:**
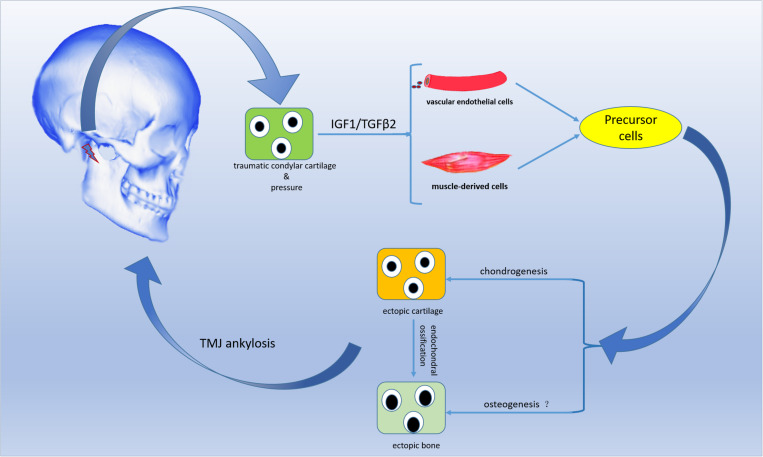
Schematic diagram of the proposed development process of THO-TMJ. Trauma in the TMJ region stimulates residual and injured chondrocytes to release IGF1/TGFβ2 when there is no articular disc (and under pressure). IGF1/TGFβ2 may induce blood endothelial cells and muscle-derived cells to differentiate into chondrocytes, followed by heterotopic ossification through an endochondral ossification process. Finally, heterotopic ossification may lead to TMJ ankylosis in the clinic. “?” indicates that precursor cells may also directly differentiate into osteoblasts, but this needs to be further investigated.

In summary, the residual and injured condylar cartilage, but not the residual subchondral bone, played a crucial role in the pathological development of THO-TMJ, and the articular disc might be an important factor inhibiting HO formation. The injured chondrocytes under a pressure condition might secrete some growth factors, such as IGF1 and TGFβ2, that stimulate precursor cells, such as endothelial cells and muscle-derived cells, to differentiate into chondrocytes or osteoblasts that contribute to THO-TMJ formation (Figure 10). However, the exact molecular relationships among trauma, the injured condylar cartilage, growth factors such as TGFβ2 and pressure, as well as precursor cells need to be explored in detail in the future.

## Data Availability Statement

The raw data supporting the conclusions of this article will be made available by the authors, without undue reservation, to any qualified researcher.

## Ethics STatement

The animal study was reviewed and approved by Ethics Committees of the Shanghai Ninth People’s Hospital, Shanghai Jiao Tong University School of Medicine, China.

## Author Contributions

YZ, JD, and GS designed the study, analyzed the data, wrote the manuscript, and approved the final version of manuscript. YZ, NO, HZ, and LC performed the experiments. All authors contributed to the article and approved the submitted version.

## Conflict of Interest

The authors declare that the research was conducted in the absence of any commercial or financial relationships that could be construed as a potential conflict of interest.
