# Supplementary material for: Stimulating Factors and Origins of Precursor Cells in Traumatic Heterotopic Ossification Around the Temporomandibular Joint in Mice
Source: Front Cell Dev Biol. 2020 Jun 18;8:445. doi: 10.3389/fcell.2020.00445 (PMC7314999; doi:10.3389/fcell.2020.00445)
Supplement: Supplementary file 1 [file Image_1.pdf]

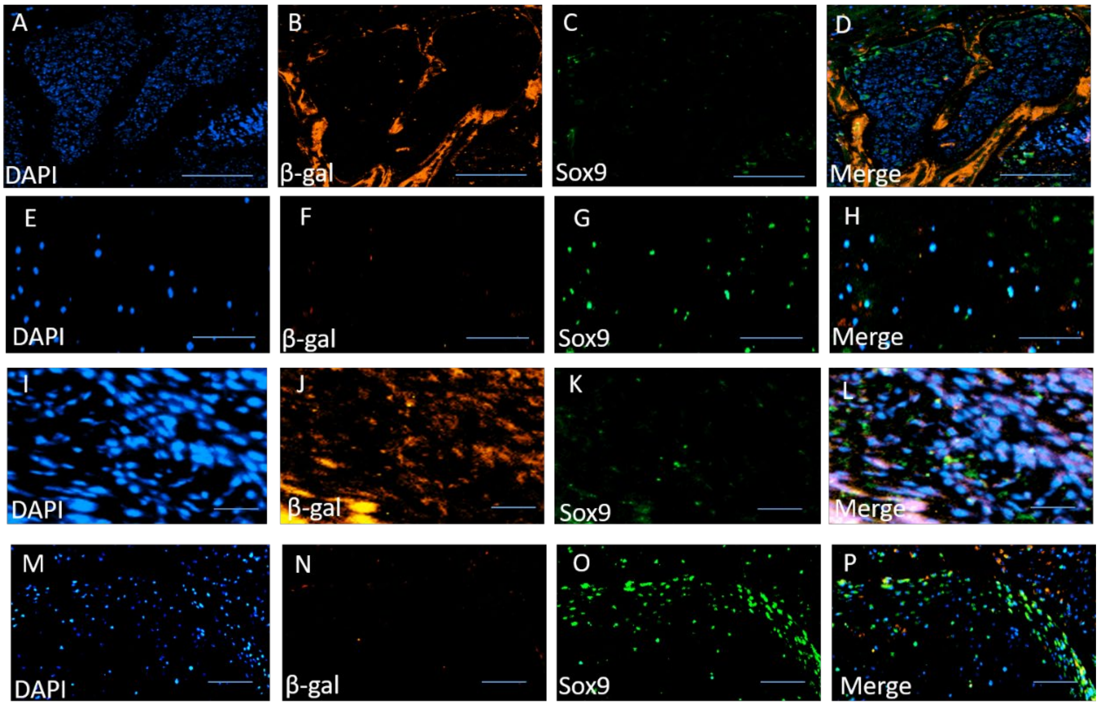

Suppl. Fig. 1. The positive and negative control groups for the lineage tracing mouse models. **(A-D)** Tie2-Cre/Lacz$^{flox/flox}$ (positive control): vascular tissue of mandibular bone marrow cavity which showed obvious β-gal expressed by Lacz but little Sox9, scale bar: 50μm. **(E-H)** Tie2-Cre/Lacz$^{flox/flox}$ (negative control): condylar cartilage of mandibular bone which has no vascular tissue and showed obvious Sox9 but little β-gal expressed by Lacz, scale bar: 25μm. **(I-L)** Ckmm-Cre/Lacz$^{flox/flox}$ (positive control): muscular tissue around condyle which showed obvious β-gal expressed by Lacz but little Sox9, scale bar: 25μm. **(M-P)** Ckmm-Cre/Lacz$^{flox/flox}$ (negative control): condylar cartilage of mandibular bone which has no muscular tissue and showed obvious Sox9 but little β-gal expressed by Lacz, scale bar: 50μm.
